# Impact of CGIAR maize germplasm in Sub-Saharan Africa

**DOI:** 10.1016/j.fcr.2022.108756

**Published:** 2023-01-01

**Authors:** Vijesh V. Krishna, Maximina A. Lantican, B.M. Prasanna, Kevin Pixley, Tahirou Abdoulaye, Abebe Menkir, Marianne Bänziger, Olaf Erenstein

**Affiliations:** a International Maize and Wheat Improvement Center (CIMMYT), ICRISAT Campus, Patancheru, 502324 Hyderabad, India; b CIMMYT, Mexico City, Mexico; c CIMMYT, Nairobi, Kenya; d International Institute of Tropical Agriculture (IITA), Bamako, Mali; e IITA, Ibadan, Nigeria

**Keywords:** CIMMYT, International Maize and Wheat Improvement Center, CRP, CGIAR Research Program, DPD, dynamic panel data, IITA, International Institute of Tropical Agriculture, GMM, generalized method of moments, NARS, National Agriculture Research System, OLS, ordinary least squares, OPVs, open-pollinated varieties, proVA, provitamin-A, QPM, quality protein maize, R&D, research-and-development, SME, small and medium enterprise, SSA, sub-Saharan Africa, CGIAR, Crop research program, Improved germplasm, Varietal adoption, Economic impact

## Abstract

This study reports on the adoption and impacts of CGIAR-related maize varieties in 18 major maize-producing countries in sub-Saharan Africa (SSA) during 1995–2015. Of the 1345 maize varieties released during this timeframe, approximately 60% had a known CGIAR parentage. About 34% (9.5 million ha) of the total maize area in 2015 was cultivated with 'new' CGIAR-related maize varieties released between 1995 and 2015. In the same year, an additional 13% of the maize area was cultivated with 'old' CGIAR-related maize varieties released before 1995. The aggregate annual economic benefit of using new CGIAR-related maize germplasm for yield increase in SSA was estimated at US$1.1–1.6 billion in 2015, which we attributed equally to co-investments by CGIAR funders, public-sector national research and extension programs, and private sector partners. Given that the annual global investment in CGIAR maize breeding at its maximum was US$30 million, the benefit-cost ratios for the CGIAR investment and CGIAR-attributable portion of economic benefits varied from 12:1–17:1, under the assumption of a 5-year lag in the research investment to yield returns. The study also discusses the methodological challenges involved in large-scale impact assessments. Post-2015 CGIAR tropical maize breeding efforts have had a strong emphasis on stress tolerance.

## Highlights

• This study estimates the impact of CGIAR maize germplasm for the period 1995–2015.
• About 60% of the new maize varieties released in the 18 study countries in Africa had CGIAR germplasm relationship.
• One-third of the maize area was under new CGIAR varieties in 2015.
• In 2015, associated annual aggregate economic benefits attributed to CGIAR maize breeding were US$0.37–0.53 billion.
• Compared to the benefits, global investment in CGIAR maize improvement was modest.

## 1 Introduction

Sustainably providing sufficient, nutritious, accessible, and affordable food supplies for a growing population of the Global South is a major challenge, which is aggravated by climate change (Connolly-Boutin and Smit, 2016). The pathways to increase food production include expanding the land area under cultivation (extensification), increasing the intensity of production per unit of land, and increasing production efficiency. As extensification of agriculture often implies unfavorable societal and environmental trade-offs (Johnson et al., 2014), sustainable intensification of existing production systems through technological change has become the focus of research-and-development (R&D) initiatives. Increased research investment and policies that enable structural changes in national and international agricultural R&D programs are essential, and it is equally important to monitor the effectiveness of these investments through rigorous impact assessments (Kubitza and Krishna, 2020). Here we examine the impact of CGIAR on improved maize germplasm with active investment and involvement of a diversity of national partners in sub-Saharan Africa (SSA) over a 21-year period (1995–2015).

Globally, maize has strategic importance as a supplier of food and feed energy and protein (Poole et al., 2021, Erenstein et al., 2022). Technological innovations, combined with an expansion in the cultivated area, have enabled farmers to realize the global maize grain production of one billion tons per annum in 2013 and maintain a consistent, positive growth rate in yield (Byerlee and Edmeades, 2021). Despite the constantly increasing production, meeting global demand for maize from food, feed, and fuel markets has remained a major challenge for the producing countries, particularly in the Global South (Erenstein et al., 2022), where agricultural systems increasingly suffer from depletion and degradation of the resource-base, frequent pest infestation, and the vagaries of climate change (Challinor et al., 2016, Abro et al., 2021, Overton et al., 2021). The production conditions are particularly challenging in SSA for maize, with the increased intensity of biotic and abiotic stress factors, soils low in organic matter and other nutrients, and limited use of external inputs. As a result, African countries contributed only 7.5% of global maize production from an area share of 20.5% during 2010–2020 (estimated using data from FAOSTAT, 2022). Unlike the leading maize producers (e.g., the USA, China, and India), the yield growth registered in Africa has also been modest (12 kg ha^−1^ y^−1^, during 2010–2020; estimated using data from FAOSTAT, 2022), with significant intra-continental heterogeneity. Against these challenges, CGIAR maize R&D has been generating and popularizing yield-enhancing, stress-resilient, and nutrient-rich varieties and improved farming practices in close collaboration between the International Maize and Wheat Improvement Center (CIMMYT), the International Institute of Tropical Agriculture (IITA), national breeding programs, the private seed sector, and other national partners (Jones-Garcia and Krishna, 2021).

Against the context of drastic institutional changes in the international agricultural R&D and increasing intensity of biotic and abiotic stress factors limiting maize production, an examination of the role of CGIAR institutions in sustaining the maize yield growth in Africa is warranted. CGIAR, formerly known as the Consultative Group for International Agricultural Research, established in 1971, is a global partnership that unites international organizations engaged in agricultural R&D to "deliver science and innovation that advance transformation of food, land, and water systems in a climate crisis" (CGIAR, 2020, p. 17). The pro-poor impacts of research conducted under the aegis of CGIAR have been previously documented (Renkow and Byerlee, 2010).

Historically, the maize breeding programs of CGIAR have been involved in developing new varieties with greater genetic yield potential (Byerlee and Edmeades, 2021, Masuka et al., 2017, Paudel et al., 2022), higher biotic and abiotic stress tolerance or resilience (e.g., drought-tolerant or DT maize; Katengeza and Holden, 2021; Prasanna et al., 2021), and enhanced nutrient content (e.g., quality protein maize or QPM; Goredema-Matongera et al., 2021; Maqbool et al., 2021) for farmers of South Asia, SSA, and Latin America. Development and dissemination of such varieties help adapt the maize production systems to climate change and to impart resistance to major biotic stresses, including diseases (e.g., maize streak virus, maize lethal necrosis, Turcicum leaf blight, grey leaf spot, tar spot complex, stalk rots, ear rots, etc.), parasitic weeds (e.g., *Striga* spp.), and insect-pests (e.g., fall armyworm, *Spodoptera frugiperda*) (Prasanna et al., 2021, Gasura et al., 2022, Kamweru et al., 2022). Since 2015, marker-assisted forward breeding and marker-assisted backcrossing have been effectively used in eastern and southern Africa by CIMMYT to introgress resistance to maize streak virus and maize lethal necrosis into diverse genetic backgrounds (Prasanna et al., 2021). Significant strides have also been made in breeding elite maize lines and hybrids with native genetic resistance to fall armyworm in Africa, based on the strong foundation of insect-resistant tropical germplasm developed by CIMMYT scientists in Mexico. These efforts are further intensified to develop and deploy elite maize cultivars with native fall armyworm tolerance / resistance and farmer-preferred traits suitable for diverse agroecologies in Africa and Asia (Prasanna et al., 2022).

Over the last three decades, the CGIAR R&D investment has shown a gradual but significant change in the regional focus. A "pivot to Africa," as described by Byerlee and Edmeades (2021), was initiated from the funding crisis that CGIAR faced in the 1990s. The African focus on CGIAR maize breeding intensified even further after the establishment of the CGIAR Research Programs (CRPs), especially CRP MAIZE. A detailed history of the evolution of the maize breeding program in CGIAR is presented by Byerlee and Edmeades (2021), which started with an optimism that due to the increased R&D investment, the African countries might be realizing a long-awaited "maize revolution." Despite the heightened Africa focus, CGIAR institutions continue to address the production constraints of smallholders in Asia and Latin America, with several maize varieties annually released in conjunction with public and private breeding institutions (Tesfaye et al., 2017, Guzzon et al., 2021).

In this study, we estimate the impact of CGIAR germplasm on enhancing maize production in 18 target countries in SSA during 1995–2015. The effect of agricultural R&D under the aegis of CGIAR institutions has been closely scrutinized. Most of the past impact evaluations indicated favorable results. In one of the earliest global impact evaluations (Evenson and Gollin, 2003), the rates of returns on CGIAR investment in crop germplasm improvement were found to be within the range of 39% in Latin America to 100% in Asia over 1965–1998. In a meta-analysis, Raitzer and Kelley (2008) showed that the benefit-cost ratios of CGIAR interventions could range from 2:1–17:1. A recent assessment reiterated the financial advantage of CGIAR research programs, indicating that investment in CGIAR R&D resulted in a benefit-cost ratio of 10:1 (Alston et al., 2022). Despite the reported significant economic gains realized from past investments, the stagnation of international funding for agricultural R&D (Zeigler and Mohanty, 2010) can be viewed only as a paradox. Given the drastic decline in CGIAR funding after 2014 (Beintema and Echeverría, 2020), the systematic documentation of the economic effects of international agricultural R&D has become even more important.

The present study updates the estimates of collaborative international maize breeding for earlier periods: 1966–1990 (Lopez-Pereira and Morris, 1994), 1966–1998 (Morris et al., 2003), and 1981–2005 (Alene et al., 2009). Our conservative assessments point toward an overall benefit-cost ratio ranging between 12:1 and 17:1 for R&D investment in CGIAR maize breeding in SSA, highlighting the economic value of increasing the public R&D investment across the Global South.

## 2 Materials and methods

### 2.1 Study area

We focused on the 18 major maize-producing countries of SSA: Angola, Benin, Cameroon, Ethiopia, Ghana, Guinea, Kenya, Madagascar, Malawi, Mali, Mozambique, Nigeria, Rwanda, Senegal, Tanzania, Uganda, Zambia, and Zimbabwe, from where data were available on varietal releases and their parentage. There was a steady increase in the aggregate maize area in these 18 countries during the study period, from 17 million ha in 1995 to 28 million in 2015 (FAOSTAT, 2021). However, maize yields in Africa remained low, with a significant inter-country variation. Among the 18 focal countries in 2015, the average maize yield ranged from 0.6 t ha^−1^ (Zimbabwe) to 3.7 t ha^−1^ (Ethiopia), and no country in the subcontinent had a yield above the global average (5.5 t ha^−1^; FAOSTAT, 2021).

For CGIAR centers, the enhancement of maize production in SSA has become a top priority since the mid-1980s (Byerlee and Edmeades, 2021). CIMMYT and IITA continuously exchanged improved maize germplasm and integrated novel tools and technologies to increase genetic gains in the stress-prone environments of the continent. CIMMYT, in particular, integrated doubled haploid technology, high-throughput, field-based phenotyping, and genomics-assisted breeding for the breeding pipelines (Chaikam et al., 2019). Since 2002, the two centers have intensified public-private partnerships to strengthen the maize seed systems in the target countries of Africa. Several third-party funded projects, such as 'Drought Tolerant Maize for Africa,' 'Stress Tolerant Maize for Africa,' 'Water Efficient Maize for Africa,' 'Improved Maize for African Soils,' and seed-sector-related investments provided a strong impetus to these R&D efforts (see https://www.cimmyt.org).

### 2.2 Estimating the contribution of CGIAR germplasm in recent maize varietal releases

Parentage or pedigree information for different improved maize varieties released between 1995 and 2015 was required to determine the contribution of CGIAR germplasm. However, the parentage of commercialized maize varieties, especially from the private sector, is often not disclosed for various reasons (Morris et al., 2003). Moreover, for a cross-pollinated crop like maize, it is not easy to calculate the percentage of germplasm contribution from different sources and provide the exact contributions of the different institutions involved in the varietal release. Therefore, we relied on the 'reported CGIAR germplasm contribution' from a purposive expert survey, especially for those varietal releases lacking exact pedigree information. A structured questionnaire was sent to public-sector agricultural research programs, including ministries of agriculture, research and extension institutes and agricultural universities, CIMMYT and IITA regional offices, and private sector scientists and managers operating in SSA, with a covering letter ensuring confidentiality. The questionnaire elicited the details at the individual variety level for the region or country, including varietal name, release year, pedigree, relation to CGIAR institutions, the main use of the variety in the country (food, feed, etc.), cultivar type (OPV, hybrid, etc.), maturity (early, late, etc.), grain color, suitable agroecology for the variety, nutritional attributes, and success rate in terms of adoption rate. The expert survey dataset was instrumental in estimating the CGIAR relationship in the varietal releases and for adoption estimation.^1^

Based on the data obtained from expert surveys and secondary sources, maize varieties were assigned to five categories based on CGIAR germplasm relationship: (1) varieties with 100% CGIAR parentage (all parents derived from CGIAR germplasm), (2) varieties with significant CGIAR parentage (50–99%), (3) varieties with some CGIAR parentage (1–49%), (4) varieties with no known/reported CGIAR parentage, and (5) varieties of unknown parentage. Because of category (5), the estimates for the contribution of CGIAR maize germplasm to varietal releases reported in this study may be cautiously approached and understood as the lower bound values and a likely underestimation of the real impact. Varieties for which only qualitative information was obtained from the experts and/or literature (e.g., some sources revealed CGIAR germplasm parentage only as "mostly," "limited," etc.) are included in the best corresponding categories of (2) or (3). The category of 'CGIAR-related varieties,' which is the focus of the present study, is generated as the aggregate of (1)-(3). For a meaningful inter-country comparison, we estimated the annual varietal release intensities, calculated as the number of maize varieties released in a country per annum during 1995–2015 divided by the area under maize in the base year (1995).

### 2.3 Estimating the on-farm varietal adoption

We gathered the adoption data from the 18 target countries for the study period from all available sources. As the foundation, we used an expert survey focusing on estimated varietal adoption for the most recent year (2015). We sent a semi-structured questionnaire to 71 maize scientists/ managers from private and public maize breeding programs in these countries in 2016 and received 43 respondents, resulting in a response rate of 61%. The dataset was further enriched with a list of improved maize varieties released during 1995–2015 from the seed catalogs, variety registers, and data collected from earlier studies, including that of Walker and Alwang (2015), peer-reviewed journal papers, and project reports. These sources also provided the pedigree information (i.e., CGIAR germplasm relationship) and the estimated area coverage of individual varieties. A list of reviewed studies is provided in Supplementary Materials S1. Of the total of 1548 variety-specific adoption data points over the study period, 27% were from published literature, 50% from unpublished project documents (especially CGIAR project, "Diffusion and Impact of Improved Varieties in Africa," DIIVA; www.asti.cgiar.org/diiva), 12% from seed registries, and 10% from CIMMYT expert surveys. The adoption figures on OPVs relied more on unpublished data and literature review, whereas those on hybrids relied on unpublished reports and seed sales registries.

Unlike the expert survey estimates, the reported adoption figures from the literature do not correspond to a single year. However, to estimate the CGIAR germplasm contribution to the varieties grown each year, we required data on annual adoption rates for individual varieties over the entire study period. The temporal adoption patterns were obtained by extrapolating data from different points in time for each released variety in use. We assumed a linear increase from zero in the year of release to the first observed average adoption rate. We assumed that the adoption rate would hold constant at the observed level for five years and then decline to zero over the next five years. The estimated diffusion rates were uniformly rescaled as needed to ensure the aggregate estimated adoption in a particular year did not exceed 100%. We acknowledge that the assumptions behind the extrapolation of temporal adoption patterns across sources are strong and also recognize that the estimates from different sources may vary in their robustness and representativeness. At the same time, the dependence on multiple sources for obtaining the adoption rates of individual varieties could result in more robust aggregate adoption estimates.

### 2.4 Estimating the impact of CGIAR germplasm

#### 2.4.1 Estimating the yield effects of CGIAR germplasm

The first step of the economic impact estimation of CGIAR germplasm on maize production in SSA is an assessment of the grain yield effects of CGIAR-related varieties. Panel data regression models with country-level fixed effects were employed to capture the effect of the change in acreage share of maize varieties with CGIAR parentage on national maize yield. National-level maize yield was used as the dependent variable, and the estimated share of maize area under the new and old CGIAR germplasm-related varieties as the key independent variables. The comparison is made amongst all alternative varieties (i.e., old/new non-CGIAR varieties and varieties without parentage information), and these varieties occupied nearly two-thirds of the maize area in the study countries in 2015.

The conventional fixed-effects models control for cross-section heterogeneity and time-invariant unobservable factors while improving the efficiency of parameter estimates. However, to precisely estimate the relationship between maize yield and CGIAR germplasm contribution in varietal development, we must also control for unobserved time-variant heterogeneity. For this purpose, we used a Dynamic Panel Data (DPD) model with a balanced dataset between 1995 and 2015. Arellano and Bond (1991) developed a Generalized Method of Moments(GMM) estimator for the DPD model using the first difference of the equation. In our case, the model was estimated as:

(1) $$\Delta \ln\left(y_{\mathrm{it}}\right)=a+\;l_{1}\Delta \ln\left(y_{\mathrm{it}-1}\right)+\;l_{2}\;\Delta \ln\left(y_{\mathrm{it}-2}\right)+\;b_{2}\Delta \mathrm{CGIA}R_{\mathrm{it}-j}+\;\sum_{k=1}^{k}g_{k}C_{\mathrm{itk}}+\;h\left(T\right)+{(e}_{\mathrm{it}}-e_{\mathrm{it}-1})$$

where $y_{\mathrm{it}}$ is the national maize yield (kg ha^−1^) in the country i in year t, derived from the FAOSTAT (2021) database. $\mathrm{CGIA}R_{\mathrm{it}}$ is the share of CGIAR-related varieties in the national maize acreage, $C_{\mathrm{itk}}$ is the set of k country-specific variables (including gross cropped area, percentage share of maize area, percentage share of irrigated area, annual rainfall, chemical fertilizer use in the country), T is the dummy variable standing for the time of observation, and $e_{\mathrm{it}}$ is the remainder disturbance that can vary over time and countries. In the variable set $C_{\mathrm{itk}}$, we included only the time variant elements that are available for the study period for all countries and have a potential effect on maize grains. All time-invariant country-specific variables will be omitted in the model estimation, and hence many policy- and market-specific variables were not suitable to include.

Our primary focus is to estimate $b_{2}$, the effects of CGIAR-related germplasm on national maize yield. Estimation of Eq. (1) requires an instrumental variable to correct for endogeneity, especially with the variable $\mathrm{CGIA}R_{\mathrm{it}}$ and for correlation between the lagged differences of the dependent variable and $e_{\mathrm{it}-1}$. Under additional assumptions, it is possible to construct an alternative GMM estimator that overcomes this problem. Here we follow Arellano–Bover/Blundell–Bond estimation (Arellano and Bover, 1995, Blundell and Bond, 1998), which is a linear DPD model with lags of the dependent and key explanatory variables. Similar models have been used by several researchers of agricultural economics to avoid endogeneity and to generate robust impact estimates (Klomp and Haan, 2013, Krishna et al., 2015). The yield effect size ($b_{2}$) is then compared with the farm-level impact of CGIAR germplasm from the literature.

In some countries (e.g., Nigeria), a significant share of farmer acreage under maize in 2014 and 2015 was cultivated with 'varieties of unknown parentage' that might or might not include CGIAR-related varieties. To verify to what extent such measurement error affects the model estimates, we have re-run Eq. (1) with a reduced sample. Observations from years when varieties with unknown parentage were greater than 10% were excluded from the model estimation.

The CGIAR adoption percentage may stand proxy for the changing breeding capabilities of the study countries, and hence, one may argue that our approach overestimates the effect size. We included the number of researchers and share of researchers working in the public sector with a 5-year lag (obtained from Beintema et al., 2012) among the explanatory variables in the regression function and compared the effect of CGIAR varieties across the two sets of regression models. An insignificant change in the CGIAR impact estimates would reject the hypothesis that the model estimates capture the effect of an increase in the breeding capacity of the study nations.

Finally, we attempt to identify the sources of the CGIAR germplasm effect, hypothesized to be dependent on the type of varieties that were replaced due to the spread of CGIAR varieties: old non-CGIAR varieties (including local varieties) or new ones. Varietal age, germplasm adaptability to the local agroclimatic conditions, genetic potential, and seed quality generate the yield effect, had the old varieties been replaced. On the other hand, germplasm adaptability and genetic potential dominate when new varieties are replaced. Delineating these factors is a challenging task. We categorized observations into two groups, based on farmer adoption of new maize germplasm (both CGIAR and non-CGIAR, released after 1994) and taking the 50% adoption rate as the cutoff. The observations from 2006 to 2015 are analyzed using regression tools to see whether there is a significant difference in the effect size across the two groups. We excluded the 1995–2005 observations for not having information on non-CGIAR varieties released immediately before (e.g., varieties released in 1994, which would qualify as a recently released modern variety during that period). While a significant difference in effect magnitude across the groups would indicate the potential causal pathways, this approach has certain limitations – a reduced sample size, the lack of accurate data on old local varieties, endogenous factors (e.g., government policies) that affect general seed sector development, CGIAR institutional performance and maize productivity, etc. A more sophisticated approach would be allowing for a heterogeneous impact of CGIAR germplasm by including a quadratic term of share of maize area under CGIAR-related varieties in the regression analysis. We anticipate the marginal effect of CGIAR varieties to be high when its diffusion is low because there will still be a significant area share with low productive varieties in the comparison group. The marginal effect is expected to diminish with increasing diffusion as the lowest-yielding varieties are gradually ousted from the agroecosystems and the control group, and the comparison is made with the well-adapted non-CGIAR varieties that survived the competition from the CGIAR varieties for the cultivated area.

#### 2.4.2 Estimating the economic surplus changes

To estimate the economic impacts of CGIAR-related germplasm through yield enhancement, we employed an equilibrium displacement model. The partial equilibrium framework has long been used in evaluating commodity-related technological progress in agriculture for impact assessments (Krishna and Qaim, 2008, Alene et al., 2018). The economic surplus changes due to intervention (for this study, CGIAR-related germplasm) were estimated based on a parallel shift in the supply curve of the commodity (for this study, maize grain). The economic benefits from CGIAR maize germplasm coupled with investments from the diversity of national partners and seed companies were estimated separately for each study country within a framework for the small open economy adapted for *ex-post* impact evaluation (Alston et al., 1995). However, since the 18 study countries taken together only represent a limited share of global foreign trade in maize: e.g., 1% of world import quantity and 0.5% of world export quantity during the second half (2006–2015) of the study period (FAOSTAT, 2021), we assume no spillover effects of the yield increase in SSA over to the global maize economy and other maize producing countries. The economic surplus attributed to CGIAR maize germplasm at the time t $\left({\mathrm{ES}}_{t}\right)$ was estimated as:

(2) $${\mathrm{ES}}_{t}=P_{t}Q_{t}K_{t}\;\left(1-0.5K_{t}\varepsilon \right)$$

where $P_{t}$ and $Q_{t}\;$are the average real-world market price of maize and the total maize production of the country in year t, respectively. For the present analysis, we used the international price of maize grain in 2015 and estimated the surplus values. The per-unit cost reduction results in a supply function shift of magnitude $K_{t}$. Finally, ε is the price elasticity of the supply of maize grains. Using these parameters, the supply shift due to CGIAR-related germplasm was calculated as:

(3) $$K_{t}=\left(\frac{\left(\Delta Y/Y\right)}{\varepsilon }-\frac{\Delta C/C}{1+\Delta Y/Y}\right).A_{t}$$

ΔY/Y and ΔC/C are the average proportional change in yield and average proportional change in the variable costs per land unit associated with technology adoption, and $A_{t}$ is the adoption rate of CGIAR-related varieties (share of maize area in the country). The net yield gain for recent CGIAR-related varieties over others (including old CGIAR-related varieties, recent and old non-CGIAR varieties, and landraces) was derived from the regression estimates. We assumed that the cultivation of CGIAR-related maize varieties did not cause a variable cost change, making ΔC/C=0 (i.e., the new CGIAR-related seed cost is the same as that of non-CGIAR varieties, and we assume no change in other input use).^2^

## 3 Results

### 3.1 Maize varietal releases and CGIAR germplasm contribution

**Fig. 1:**
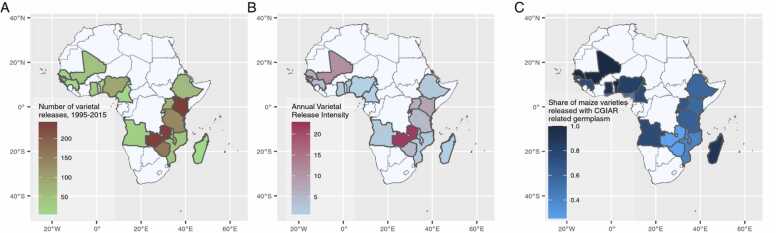
Maize varietal release indicators and share of CGIAR-related maize germplasm, 1995–2015 in the study countries in SSA. Notes: **A**. Total number of maize varieties released in the country during 1995–2015. **B**. Annual maize varietal release intensity (average number of maize varietal releases in a country per annum during 1995–2015 divided by the national area under maize in the base year, 1995). **C**. Share of maize varieties released with CGIAR-related germplasm (0–100%).

The details of improved maize varieties released during 1995–2015 and CGIAR germplasm contribution in their parentage are depicted in Fig. 1 (and Appendix I). During the study period, a total of 1345 maize varieties were released in the 18 focus countries. The average number of annual varietal releases varied from < 1 per annum in Benin, Cameroon, Guinea, Madagascar, and Senegal to 11–12 per annum in Kenya and Zambia (Fig. 1A). The release intensity was the highest for Rwanda (23 varieties per million ha) and Zambia (22 varieties) and the lowest for Nigeria and Cameroon (0.83 varieties) (Fig. 1B). Among all the maize varieties released between 1995 and 2015, about 60% had CGIAR inbred lines directly in their pedigree or had parental lines with some CGIAR origin (Fig. 1C). About 78% of CGIAR varieties with known parentage were developed by the public sector, 5% under public-private partnerships, and the rest (17%) solely by the private sector. In contrast, most (88%) non-CGIAR varieties were developed by private companies. We suspect a certain degree of under-reporting of varieties with CGIAR parentage, especially in the large maize producer countries, such as Kenya, Ethiopia, Mozambique, Tanzania, etc., where the share of varieties with unknown parentage was high. In these countries, a large share of varieties was developed as crosses of CGIAR and non-CGIAR germplasm (e.g., 42% in Kenya), pointing toward significant collaborative R&D efforts of the national public and private sector partners. However, all uses of CGIAR germplasm (and hence their presence in the parentage) might not always be (fully) acknowledged by the partner institutions.

A negative relationship (r = –0.70, p < 0.01) is observed between the number of overall maize varietal releases and the share of CGIAR-related releases. The share of CGIAR-related releases was higher in countries with fewer aggregate releases, while the share decreased as the number of total releases increased. For example, almost all maize varieties released between 1995 and 2015 in Senegal and Benin had CGIAR parentage, although the annual varietal release was less than 1.0. Nonetheless, even in countries with high varietal release intensity, the share of CGIAR-related releases was not substantially low. A possible reason behind this pattern could be the presence of an active and well-developed private seed sector and the existence of strong public-private partnerships in the above-mentioned countries.

Although most new varieties released during 1995–2015 were hybrids, the share of hybrids was significantly lower (64.5%) among CGIAR-related varieties than among non-CGIAR varieties (73.5%) (Table 1). The country-specific adoption of new hybrids (Supplementary Materials S2) further shows that hybrid maize adoption had been marginal in countries with less-developed seed markets (e.g., Mali, Benin, Senegal, etc.), where most of the maize varieties were derived from CGIAR germplasm. These patterns validate the research focus of CGIAR and partners on developing affordable OPVs for poor farmers where seed markets are less developed.

**Table 1:** Characteristics of maize varieties released during 1995–2015 in 18 study countries.

	Number of maize varietal releases		
	Open-pollinated varieties (OPVs)	Hybrids	Overall
Non-CGIAR	152 (11.1%)	422 (30.7%)	574 (41.8%)
CGIAR	284 (20.7%)	517 (37.6%)	801 (58.3%)
Overall	436 (31.7%)	939 (68.3%)	1375 (100.0%)

Notes: Figures in parentheses show the percentage share of all varieties with known parentage and released during the study period.

The measure of row-column association, Pearson χ^2^, is 12.44 (p ≤ 0.01).

### 3.2 Farmers' adoption of CGIAR-related maize varieties

**Fig. 2:**
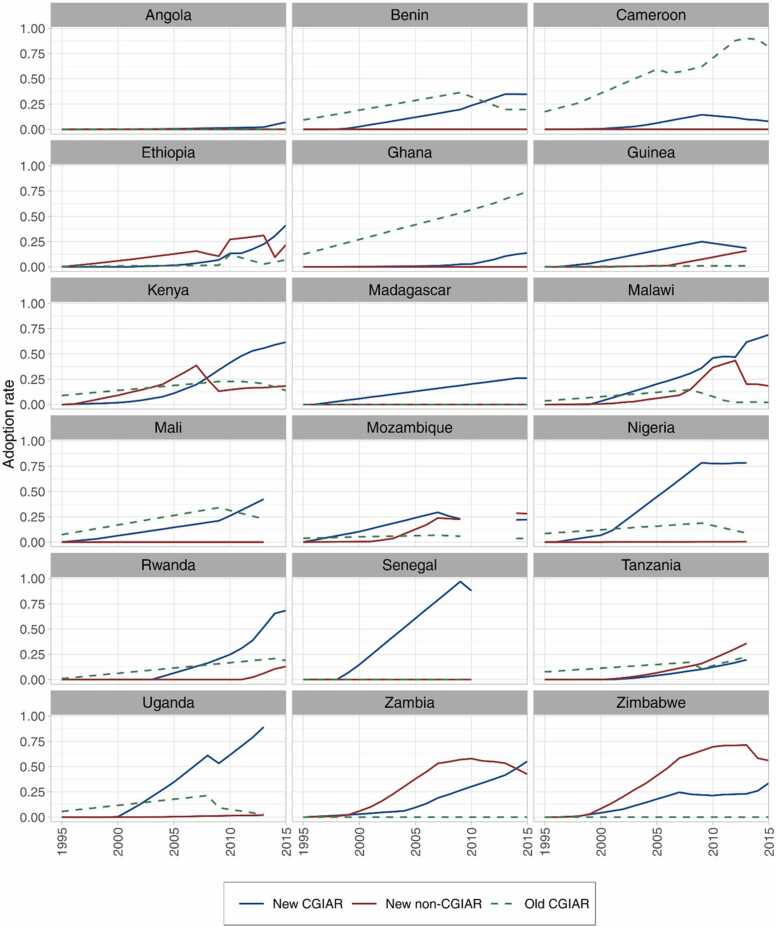
Estimated farmer use of CGIAR-related (new and old) and non-CGIAR (new) maize varieties in each of the study countries in SSA. Notes: 'New' are varieties released during the study period (1995–2015), and 'old' (CGIAR) are those released before 1995. No estimates are shown for the years when new varieties with unknown parentage are > 10% of the national share.

The estimated share of area under improved maize varieties released between 1995 and 2015 in the target countries increased progressively over the study period, reaching 69% by 2015. Fig. 2 presents the temporal adoption of new and old CGIAR-related varieties by country over the 21-year period. The adoption of new, improved maize varieties increased at a rate of 3.5% points per annum overall. The adoption of CGIAR-related maize varieties (new and old) was faster than those of the non-CGIAR varieties (2.2% points vs. 0.9% points, respectively).

The maize area in 2015 could be divided into three groups that are similar in size, viz. 'old' varieties, new CGIAR varieties, and the combination of new non-CGIAR varieties and unknown. The first group of 'old' varieties includes local varieties, landraces, and improved maize varieties released before 1995, whether related to CGIAR or not. The area cultivated with these varieties was estimated to be about 8.7 million ha (31% of the total maize area) in 2015. Of this, 3.3 million ha were covered with old CGIAR-related varieties released before 1995. In Cameroon and Ghana, the old CGIAR varieties were dominant in the farmers' fields even in 2015. The second group includes the new CGIAR-related improved maize varieties released after 1995 that covered about 34% of the total maize area in 2015, representing 9.5 million ha. These varieties are popular among smallholder farmers of various maize-growing countries in SSA, including Malawi, Kenya, Nigeria, Uganda, and Senegal. The final group includes the new non-CGIAR varieties cultivated in 14% of the maize area in 2015 and varieties having no known parentage (21%) and accounted for 9.8 million ha. The area under non-CGIAR varieties was particularly high in Zimbabwe and Zambia in 2015.

### 3.3 Economic impacts of recent CGIAR-related maize varieties

In this section, we examine whether the adoption of new CGIAR germplasm-related varieties generated a significant yield effect compared to other varietal alternatives for maize cultivation in SSA. The regression model estimates from the complete dataset (n = 378) and the reduced dataset (n = 357, excluding years when varieties with unknown parentage exceeded 10% of maize area) are presented in Table 2. All models showed that the new CGIAR germplasm-related varieties released after 1995 had a significant yield effect, although the magnitude of impact varied in size. Under the Ordinary Least Squares (OLS) framework, a 1% increase in the area under cultivation of these varieties increased the average annual national grain yield by 0.41% after controlling for other explanatory variables. The Fixed Effects and DPD models estimated effect sizes of 0.39% and 0.27%, respectively. When the reduced dataset was used for analysis, the OLS estimates increased to 0.57%, while the Fixed Effects and DPD model estimates were 0.40% and 0.29%, respectively. Adoption of old CGIAR-related maize germplasm had a significant effect on yield only in the OLS models (0.48%).

**Table 2:** Effect of farmer adoption of CGIAR germplasm on maize yield (kg ha^−1^) at the national level in the study countries in SSA.

	Whole sample			Reduced sample (excluding years when varieties with unknown parentage were >10%)		
	OLS	Fixed Effects	DPD	OLS	Fixed Effects	DPD
***Effect during the entire study period (1995–2015)***						
Estimated adoption of new CGIAR varieties, released in 1995 or later (share of national maize area, 0–1)	0.409^***^ (0.109)	0.389^***^ (0.109)	0.265^***^ (0.087)	0.565^***^ (0.085)	0.400^***^ (0.121)	0.289^***^ (0.098)
Estimated adoption of old CGIAR varieties, released before 1995 (share of national maize area, 0–1)	0.415^***^ (0.119)	-0.144 (0.176)	0.143 (0.111)	0.483^***^ (0.112)	-0.117 (0.192)	0.112 (0.123)
Other controls used	Yes	Yes	Yes	Yes	Yes	Yes
Number of observations	378	378	342	357	357	321
F statistics / Wald Chi^2^	29.38^***^	19.36^***^	445.00^***a^	34.10^***^	18.27^***^	493.62^***a^
***Effect during 2006–2015***						
Estimated adoption of new CGIAR varieties, released in 1995 or later (share of national maize area, 0–1)	0.413^***^ (0.130)	0.400^**^ (0.162)	0.239^**^ (0.124)	0.504^***^ (0.132)	0.547^**^ (0.262)	0.345^**^ (0.153)
Estimated adoption of old CGIAR varieties, released before 1995 (share of national maize area, 0–1)	0.308^**^ (0.154)	0.045 (0.288)	0.257 (0.159)	0.272* (0.160)	0.196 (0.330)	0.285 (0.180)
Other controls used	Yes	Yes	Yes	Yes	Yes	Yes
Number of observations	180	180	180	159	159	159
F statistics / Wald Chi^2^	12.50^***^	5.21^***^	186.92^***a^	15.71^***^	4.55^***^	209.48^***a^

Notes: The dependent variable is the log-transformed annual national maize yield (kg ha^−1^). ***: p ≤ 0.01, **: p ≤ 0.05, *: p ≤ 0.10. ^a^ Wald Chi2 statistic. OLS stands for Ordinary Least Squares and DPD for Dynamic Panel Data model.

We further tested whether the flow of CGIAR varieties stands proxy for the national capacity of maize breeding programs by including the number of research staff working in agricultural R&D and the share of public sector researchers with a five-year lag. The results are shown in Appendix II. Not only were the additional explanatory variables insignificant in the panel data models, but the effect size of CGIAR germplasm also did not change significantly from the main analysis.

**Fig. 3:**
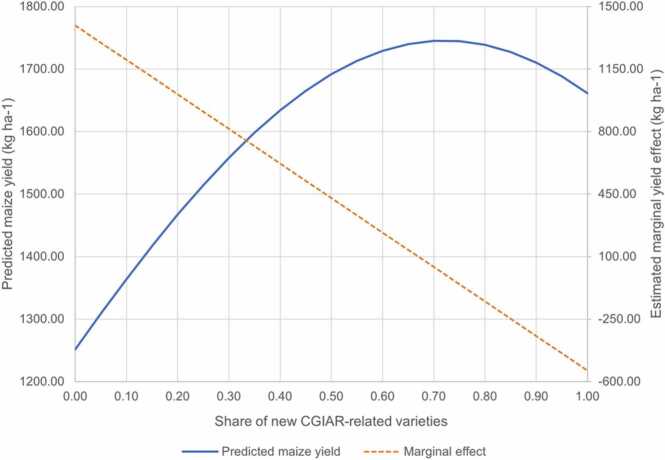
Estimated effect of new CGIAR-related maize varieties in SSA. Notes: The 'new' are the varieties released during the study period (1995–2015). The estimated values are derived from the fixed effects model with a quadratic term of the variable "Estimated adoption of new CGIAR varieties, released in 1995 or later (share of national maize area, 0–1)". The associated regression model is provided in Supplementary Materials (S3).

The sources of the yield effects of CGIAR-related varieties were identified by running regression models for 2006–2015 after grouping the observations with respect to the level of diffusion of new maize varieties. The regression estimates are provided in Appendix III. The effect magnitude was positive and statistically significant when the diffusion of new maize varieties was less than 50% and small and insignificant in the Fixed Effects model when the diffusion was 50% and above. While one may attribute the lack of statistical significance in the latter group to the reduced sample size, the pronounced yield effect of CGIAR germplasm in countries with nascent seed sectors and R&D institutions is verified by the regression analysis (Fig. 3). When a quadratic term of share of CGIAR variety adoption was introduced in the model, the marginal effect was shown declining with the diffusion of CGIAR varieties: 1300 kg ha-1 when the diffusion rate is 5%, zero yield effect at 72% diffusion rate, and negative above that. However, only a small share (<5%) of observations had the diffusion rate of CGIAR varieties above 72% of the national maize area, and the positive effect of CGIAR maize holds for 95% of observations. Together with the findings presented in Section 3.1, these two analyses provide ample empirical support and evidence for the high impact of CGIAR-related germplasm in countries with less developed seed markets.

The aggregate impact of CGIAR-related germplasm in SSA was estimated for 2015. There is a possibility that the yield effect could vary over time as new breeding technologies are constantly introduced, new partnerships are evolved, and new traits (e.g., drought tolerance) are given emphasis in maize breeding. We re-estimated the regression models using data from the 2006–2015 period (n = 180 for the whole sample and 159 for the reduced sample) and found only a minor change in the estimated effects in comparison to using the entire dataset (Table 2). However, the estimated impact increased in magnitude in both Fixed Effects and DPD models when the reduced dataset was used. We consider the DPD model the most robust and use the corresponding estimates for our aggregate impact estimation. We thereby use a conservative yield impact size of 0.24 (24%) for CGIAR-related varieties (Scenario I, based on the DPD model using the complete dataset for 2006–2015). Economic surplus values were also calculated under an alternative scenario with a more liberal impact magnitude of 0.35 (35%; Scenario II, based on the DPD model estimated using the reduced dataset for 2006–2015). The validity of these impact magnitudes can be examined through a review of the literature on drought-tolerant (DT) maize in Africa.^3^ Abdoulaye et al. (2018) and Martey et al. (2020) reported the yield effect of DT maize at + 33% in Nigeria and Ghana. Amondo et al. (2019) and Simtowe et al. (2019) estimated the impact of the same technology for maize cultivation at + 15% in Zambia and Uganda, respectively. Impacts of higher magnitudes were reported by Lunduka et al. (2019). In these micro-level studies, however, the reference group against which the impact was estimated includes both CGIAR and non-CGIAR non-DT (old and new) varieties. Hence the impact of new CGIAR-related varieties that we estimate is expected to be slightly higher than the DT maize impact figures.

The results of the equilibrium displacement model to quantify the aggregate impact of CGIAR-related germplasm in SSA are presented in Table 3. The same table also contains country-specific information on the aggregate maize area, production, adoption of new CGIAR-related varieties, and value of economic benefits under two scenarios for 2015. Under the conservative Scenario I of a 24% yield effect, the annual benefits for 2015 accrued to US$1.1 billion. There was significant inter-country variation, with about 85% of the benefits accrued to six countries, viz. Ethiopia, Kenya, Malawi, Nigeria, Uganda, and Zambia that represented 52% of the maize area of the 18 countries. Inter-country inequality was more pronounced in Western and Central Africa than in Eastern and Southern Africa, with a single country (Nigeria) accounting for most of the economic benefits. Under Scenario II (of a 35% yield effect), the economic surplus estimate increased to US$1.6 billion. There was no marked change in inter-country inequality.

**Table 3:** Estimated economic surplus from CGIAR's maize improvement efforts in the study countries in SSA, 2015.

Country	Maize area (million ha) in 2015	Maize production (million t) in 2015	Estimated adoption of new CGIAR-related maize varieties (share of national maize area, 0–1) in 2015	Estimated economic surplus in 2015 (million US$ p.a.), with	
				assumed effect size of 0.24 (Scenario 1)	assumed effect size of 0.35 (Scenario 2)
Angola	1.67	1.88	0.07	8.98	13.05
Benin	1.00	1.29	0.35	29.05	41.53
Cameroon	1.19	2.07	0.08	10.87	15.78
Ethiopia	2.11	7.88	0.41	210.68	299.89
Ghana	0.88	1.69	0.14	15.60	22.57
Guinea	0.58	0.73	0.12	5.94	8.61
Kenya	2.10	3.83	0.62	148.46	208.57
Madagascar	0.20	0.33	0.26	5.64	8.10
Malawi	1.68	2.78	0.69	119.36	166.87
Mali	0.90	2.28	0.03	4.31	6.28
Mozambique	1.57	1.26	0.22	18.45	26.56
Nigeria	6.77	10.56	0.41	278.42	396.45
Rwanda	0.24	0.37	0.68	15.77	22.05
Senegal	0.20	0.3	0.51	9.88	13.98
Tanzania	3.79	5.9	0.09	34.52	50.10
Uganda	1.13	2.65	0.68	112.47	157.33
Zambia	0.86	2.62	0.55	91.99	129.77
Zimbabwe	1.11	0.64	0.34	14.16	20.25
*Overall*	*27.98*	*49.06*	*0.34*	*1134.57*	*1607.75*

Notes: Estimated economic surplus represents the benefits associated with the adoption-induced production increase.

Supply elasticity assumed is + 0.60.

Effect size denotes ΔY/Y in Eq. (3).

The CGIAR germplasm effect is a joint product of the partnership between the National Agriculture Research Systems (NARS), the private sector, and CGIAR. Ideally, we would estimate the returns to the three jointly, but the missing cost (investment by NARS and private sector) data limits our ability to do so. We, therefore, assume that the CGIAR germplasm effect is attributed equally to the partnership between the NARS, the private sector, and CGIAR (i.e., 33.3% each).^4^ The corresponding CGIAR effect alone will then vary from US$ 374.4 million (Scenario I) to US$ 530.6 million (Scenario II) for 2015.

CGIAR investment in maize improvement was US$30 million in 2015 at the global level (Appendix IV), albeit the largest share was allocated to SSA. Corresponding estimates from other institutions involved in maize breeding, including NARS and the private sector, are not available. Considering the investment in global CGIAR maize improvement as the research cost, even the lower-bound annual disaggregated CGIAR benefit estimated for 2015 was US$ 374.4 million, representing a strong return (12:1) on the annual CGIAR investments. However, such a comparison should be made with caution as the cost and benefit figures are not directly comparable. Crop breeding is a pipeline with continuous annual investments and annual returns, but there is a significant lag between the investment cost and the returns in agricultural R&D (Alston et al., 2022). The current benefits reflect past investments, and current investments generate results in the future. In Appendix V, we provide a range of benefit-cost ratios derived under the assumption of 3-year, 5-year, and 10-year lags in research investment and 25%, 33.3%, and 75% allocation of total benefits to the CGIAR maize breeding program estimated under Scenarios I and II. The benefit-cost ratios were 12:1 for Scenario I and 17:1 for Scenario II, under the base assumptions of a 5-year lag in the research investment and 33.3% benefit allocation to the CGIAR maize breeding program to realize the observed yield changes. The benefit-cost ratios increased with further lags. Had we taken a 10-year lag in the R&D investment, the ratios would have become 19:1 and 27:1 in Scenarios I and II, respectively, because of the low research investment during the pre-CRP period. The estimated economic benefits never fell below 8:1 under any of the assumptions and scenarios.

The recent advances in the CGIAR maize breeding program in the selected SSA countries are provided in Supplementary Materials S4. There we present the evidence from the literature for the benefits of stress-tolerant varieties released in collaboration between CGIAR, national breeding programs, and the private seed sector to stabilize maize production, reduce downside risk, and improve livelihoods in tropical and subtropical production environments of SSA.

## 4 Discussion

Smallholder maize farmers across Africa face many production constraints, including the increased frequency of occurrence of abiotic and biotic stresses and lack of access to improved seeds and other inputs (Prasanna et al., 2021). During the study period, CGIAR-NARS collaborative maize breeding strongly focused on developing improved maize varieties with stress tolerance and enhanced nutritional quality and delivering them in collaboration with the national and private seed sector partners, targeting critical benefits in sustaining rural livelihoods (Cairns and Prasanna, 2018, Prasanna et al., 2021). The present study estimated that CGIAR's involvement in maize germplasm improvement generated an economic surplus of US$ 0.37–0.53 billion in 2015 across the 18 study countries in SSA. The annual R&D investment at its peak was less than one-tenth of the estimated benefits. The observed significant inter-country variability in the magnitude of the impact of CGIAR germplasm could arise from differential varietal release rates and farmer adoption of new CGIAR-related varieties, and the status of development of the domestic seed sector. Zambia and Cameroon provide examples of the two extreme scenarios. Zambia's high varietal release intensity was underpinned by a vibrant private seed sector, fostered through the government's liberalization policies and a large-scale seed subsidy program (Blekking et al., 2021). The same study also concluded that "the seed subsidy program has institutionalized hybrid-maize seeds as a key component for programs aimed at alleviating rural poverty and agricultural development in the country." On the other hand, in Cameroon, a formal proprietary seed sector had historically been missing, where government agencies and farmers remained the primary seed producers (Awotide and Tontsa, 2011).

In most of the target countries in the second decade of the study, a pronounced increase in the rates of annual releases and farmer adoption was observed for the varieties having CGIAR germplasm. These patterns align with a substantial increase in available resources to support the R&D on stress-tolerant cultivars, which demonstrated superior performance over the existing varieties (Cairns and Prasanna, 2018, Byerlee and Edmeades, 2021, Prasanna et al., 2021). Especially during the second decade of the study, the number of released varieties containing CGIAR germplasm was substantially greater than the number of released non-CGIAR varieties. The effectiveness of CGIAR varieties in increasing the national yields was also higher in the last decade, as indicated by the regression analysis.

One may note that the aggregate benefit estimates reported in this paper reflect the increase in grain yield alone. While yield improvement is indeed a key performance indicator, several other less-easily quantifiable performance indicators, such as access to improved seeds and inputs, better crop management practices, etc., add to the estimated impact of international R&D in maize breeding. CGIAR efforts typically complement public and private sector R&D investments in developing and disseminating improved tropical and subtropical maize germplasm with key traits of interest to smallholder farmers, besides developing the capacity of the NARS and small and medium enterprises (SMEs). During the study period, biofortified or nutritionally enriched maize varieties, such as provitamin-A (proVA) enriched maize and quality protein maize (QPM), started to gain importance in the research pipeline (Prasanna et al., 2020). Although the efficacy of biofortified maize has been demonstrated for QPM and proVA-enriched maize (Gunaratna et al., 2010, Gannon et al., 2014), their impacts on the nutritional status and health of target populations remain difficult to quantify (Byerlee and Edmeades, 2021).

An assessment of the contribution and impact of CGIAR-related improved maize varieties should also consider yield stability in the stress-prone agroecologies. Besides aiming to increase grain yield under optimal environments, CGIAR maize improvement programs in SSA focus on providing yield stability through increasing abiotic and biotic stress tolerance, thereby contributing to the reduced downside risk of climate-induced effects on smallholder farmers. Drought tolerance was one of the primary traits pursued in CGIAR-NARS collaborative maize breeding efforts targeted at SSA since the mid-1990s, especially for stress-prone agroecologies. The effect of drought-tolerant varieties in ensuring yield stability through the reduction of unforeseen variance and downside risk has substantial welfare implications (Kostandini et al., 2013). As Byerlee and Moya (1993) highlighted, yield maintenance could contribute more gains to farmers than improved yield potential. Because such outcomes were not accounted for in the estimation, the reader may consider our economic surplus estimates as conservative, requiring revision through more advanced impact assessment studies in the future.

A narrow yield focus also ignores the broader systemic impacts of maize germplasm improvement. Enhanced yield and resilience make maize more competitive and attractive vis-à-vis other land uses. The area under maize cultivation is rapidly expanding in many parts of the developing world, primarily due to its relative profitability and better access to output markets over other crop options (Jakobsen, 2019). Area expansion could have positive impacts on farm profitability and farmer economy but might also create undesirable environmental and social effects. The nature and magnitude of these impacts will differ across maize production regions, calling for more micro-level socioeconomics and systems research. Instead of a single evaluation at the continental level, several coordinated country-specific case studies could provide more robust estimates and insights for policymakers.

A crucial limitation of our varietal adoption estimates, as for most global evaluations of agricultural research investments (Manyong et al., 2003, Kleinwechter et al., 2015, Lantican et al., 2016), is the (partial) dependence on expert surveys. Nationally representative data on varietal adoption are rarely available in lower-income countries, and it is expensive, time-consuming, and complex to carry out primary data collection from farmers or seed dealers across different study nations. While expert surveys remain as the only practical option to obtain information from multiple countries on varietal use, one may question the dependability of the derived estimates. Furthermore, getting a sufficient number of (reliable) experts in order to obtain regionally disaggregated data on varietal adoption is a challenging task. As per the recent studies using DNA fingerprinting, the adoption figures estimated using farmers' recall or expert opinion could provide a conservative estimate of the adoption of CGIAR-related varieties. A DNA fingerprinting study in Ethiopia showed that CGIAR-related maize germplasm was grown by 63% of maize-growing households in 2019, whereas self-reported data from farmers would have underestimated the adoption by 15% (Kosmowski et al., 2020). It may currently be practically impossible to conduct nationally representative and comparable studies with DNA fingerprinting across all lower-income countries simultaneously and regularly. However, supporting micro-level varietal adoption studies in selected intervention countries/regions using DNA fingerprinting would be valuable for obtaining more robust information on farmers' adoption of varieties and would help R&D institutions and programs better ameliorate different constraints in the seed value chains.

## 5 Conclusions

The estimated economic benefits of CGIAR investments in maize germplasm improvement indicate that the return to investment exceeded 12:1 in 2015. These estimates are generated after accounting for the *sine qua non* contributions of R&D partners, in particular national breeding programs and the private seed sector. This finding is similar to that of the recent impact evaluation of CGIAR R&D programs that reported a median benefit-cost ratio of 12:1 for maize R&D investment in CGIAR and NARS stream for the period 1972–2016 (Alston et al., 2022). These findings highlight that the maize breeding and seed systems work undertaken by CGIAR, together with public and private sector partners, has built strong momentum in the diffusion of improved germplasm in SSA. Our analysis also demonstrates that farmers in SSA countries can gain considerably when they obtain access to the products of international agricultural R&D.

The widespread positive impact of CGIAR investment on maize germplasm improvement masks a significant heterogeneity of the effect of varietal release intensity, farmer adoption, and resulting economic surplus across the study countries. The annual varietal release intensity during 1995–2015 ranged from < 1 variety per million ha in Cameroon and Nigeria to 22 varieties in Zambia. CGIAR institutions have been playing a particularly key role in countries where the private seed sector is relatively inactive. In about five countries – Uganda, Rwanda, Malawi, Kenya, and Zambia – the adoption of new CGIAR-related varieties was above 50% of maize area in 2015. Naturally, the estimated aggregate benefits were high in these countries. At the same time, in several other countries, the adoption of new CGIAR varieties was low (<10%), hence also the aggregate impacts. Since the adoption of new non-CGIAR varieties was also low in these countries, the weak seed distribution and extension systems could be the root cause. Historically, the CGIAR maize program has been investing primarily in developing improved germplasm, whereas complementary research on the seed value chains and adoption pathways for targeted dissemination of new varieties has received only modest support. Increased research attention and budgetary support for developing improved technology dissemination frameworks and addressing the institutional bottlenecks preventing smallholders from adopting improved germplasm are essential for even greater sustainable and equitable impacts.

## Declaration of Competing Interest

The authors declare that they have no known competing financial interests or personal relationships that could have appeared to influence the work reported in this paper.

## Figures and Tables

**Table:** 

	Annual varietal release (number) during 1995–2015	Annual varietal release intensity (number/million ha)^#^	CGIAR-parentage classes				
			Non-CGIAR	1–49% CGIAR	50–99% CGIAR	100% CGIAR	Unknown parentage
Angola	1.14	1.90	0.08	0.21	0.04	0.67	0.00
Benin	0.95	1.94	0.00	0.00	0.00	1.00	0.00
Cameroon	0.33	0.83	0.00	0.29	0.14	0.57	0.00
Ethiopia	2.95	2.02	0.16	0.03	0.18	0.44	0.19
Ghana	1.76	2.56	0.05	0.00	0.00	0.95	0.00
Guinea	0.57	3.60	0.17	0.08	0.08	0.67	0.00
Kenya	11.52	8.01	0.15	0.03	0.39	0.26	0.17
Madagascar	0.24	1.30	0.00	0.00	0.40	0.60	0.00
Malawi	4.52	3.68	0.59	0.08	0.08	0.24	0.00
Mali	2.33	11.26	0.00	0.00	0.00	1.00	0.00
Mozambique	2.05	1.90	0.30	0.05	0.09	0.33	0.23
Nigeria	4.48	0.82	0.09	0.10	0.02	0.80	0.00
Rwanda	1.14	22.86	0.08	0.04	0.21	0.63	0.04
Senegal	0.67	6.81	0.00	0.00	0.00	1.00	0.00
Tanzania	6.57	4.80	0.15	0.02	0.12	0.51	0.20
Uganda	3.71	6.50	0.08	0.08	0.21	0.49	0.15
Zambia	11.43	21.97	0.70	0.01	0.05	0.21	0.03
Zimbabwe	7.67	5.15	0.66	0.05	0.14	0.14	0.00

^#^National maize area in 1995 is used as denominator.

**Table:** 

	Whole sample			Reduced sample (excluding years when varieties with unknown parentage are >10%)		
	OLS	Fixed Effects	DPD	OLS	Fixed Effects	DPD
Estimated adoption of new CGIAR varieties, released in 1995 or later (share of national maize area, 0–1)	0.268^**^ (0.132)	0.385^***^ (0.118)	0.176^***^ (0.098)	0.435^***^ (0.102)	0.420^***^ (0.133)	0.217^***^ (0.110)
Estimated adoption of old CGIAR varieties, released before 1995 (share of national maize area, 0–1)	0.428^**^ (0.185)	-0.241 (0.244)	0.186 (0.159)	0.539^***^ (0.163)	-0.142 (0.262)	0.263 (0.170)
Researchers, total (FTEs per 100,000 farmers) with a 5-year lag	-0.017^**^ (0.007)	-0.010 (0.013)	0.005 (0.010)	-0.025^***^ (0.007)	-0.016 (0.013)	-0.002 (0.010)
Researchers from government sector (share of total FTEs, %) with a 5-year lag	0.008 (0.003)	0.010^**^ (0.004)	-0.002 (0.003)	0.001 (0.003)	0.014^**^ (0.006)	-0.002 (0.003)
Other controls used	Yes	Yes	Yes	Yes	Yes	Yes
Number of observations	303	303	277	285	285	256
F statistics / Wald Chi^2^	11.11^***^	13.80^***^	309.00^*** a^	13.33^***^	13.50^***^	321.24^*** a^

*Notes*: The dependent variable is the log-transformed annual national maize yield (kg ha^−1^). ^***^: p ≤ 0.01, ^**^: p ≤ 0.05. ^a^ Wald Chi2 statistic. OLS stands for Ordinary Least Squares, and DPD for Dynamic Panel Data model. Due to data unavailability, the observations from Cameroon and Angola are not included in the analysis.

**Table:** 

	When the adoption of new varieties is < 50% maize area		When the adoption of new varieties is ≥ 50% maize area	
	OLS	Fixed Effects	OLS	Fixed Effects
Estimated adoption of new CGIAR varieties released in 1995 or later (share of national maize area, 0–1)	1.462^***^ (0.267)	1.662^***^ (0.503)	0.544^**^ (0.265)	0.034 (0.243)
Estimated adoption of old CGIAR varieties released before 1995 (share of national maize area, 0–1)	0.092 (0.122)	0.202 (0.370)	3.017^***^ (0.680)	-0.623 (0.935)
Other controls used	Yes	Yes	Yes	Yes
Number of observations	97	97	83	83
F statistics	21.23^***^	3.34^***^	11.79^***^	1.54

Notes: The dependent variable is the log-transformed annual national maize yield (kg ha^−1^). New varieties include CGIAR-related, non-CGIAR, and varieties with unknown parentage, released after 1995. ^***^: p ≤ 0.01, ^**^: p ≤ 0.05. OLS stands for Ordinary Least Squares regression model. Due to the small sample size, the DPD models could not be estimated.

**Table:** 

*Source*: Authors' calculation with maize improvement R&D expenditures (including related seed systems/production research) estimated as 75% of CGIAR total maize program expenditures for CIMMYT Global Maize Program and IITA Maize Research Program. All costs are nominal/current prices.

**Table:** 

Time lag on the research investment (years) to yield benefits	Corresponding R&D investment as of 2015 (million USD per year)*	Benefit-cost ratio at 1/3rd benefit sharing [25–75% sharing]	
		Sc. I	Sc. II
3	30.1	12.4 [9.4–28.3]	17.6 [13.4–40.1]
5	31.9	11.8 [8.9–26.7]	16.6 [12.6–37.8]
10	19.5	19.2 [14.5–43.5]	27.2 [20.6–61.7]

Notes: *Assuming a 5% interest rate on the invested amount, the CRP MAIZE R&D investment data (shown in Appendix IV) from 2012 (3-year lag), 2010 (5-year lag), and 2005 (10-year lag) were converted following the compound interest assumption: *value in 2015 = value of the investment in nominal prices x (1+ interest rate)*^*time-lag in years*^.

The gross benefit at 33.3% benefit sharing [25–75% sharing] is 374.4 [283.6–850.9] million USD under Sc. I and 530.6 [401.9–1205.8] million USD in Sc. II in 2015.

## Data Availability

Data will be made available on request.

## Footnotes

1 There has been relatively limited technology monitoring in relation to maize breeding programs in SSA, making information on varietal release and farmer adoption of individual varieties not easily available or limited in scope. At the same time, data collection from maize breeders and project managers was time-consuming and slow. Furthermore, there were several inconsistencies in the data initially collected, especially with respect to the adoption figures. The reiterative development of the database and methodological approach took about three years (2016–2018), and statistical analysis was initiated in 2019.

2 The strong assumption of no cost increase is one of the limitations of our partial equilibrium framework estimates, which was included due to a lack of sufficient empirical evidence to assume otherwise. More socioeconomic studies are required to assess the effect of CGIAR germplasm on the production process at the farm-level, especially on external input use. Several of the CGIAR inventions are risk-reducing, and the increased certainty of production could generate a behavioral change in farmers to spend more on inputs.

3 The documented impact of DT maize from the literature is taken in particular for validating the observed effects of CGIAR germplasm, because DT is a technology developed mainly by the CGIAR institutions in SSA during the study period (Bänziger and Araus, 2007, Cairns and Prasanna, 2018). DT maize also has been the focal point of much of the CGIAR’s SSA work in the recent past, resulting in increased varietal releases and promotion. Furthermore, there has also been an increase in the documentation of varietal use with respect to DT technology. On the other hand, innovations for genetic yield potential enhancement (e.g., conventional hybrids) are developed by both CGIAR and non-CGIAR institutions, and not many have compared the effects of CGIAR and non-CGIAR germplasm in the micro-level studies.

4 We opted to give equal weight to the three entities for the simple reason that each play a key contribution – *sine qua non* – toward the final impact. Severe associated data limitations across the 18 countries during the study period limit the prospects of coming up with a more grounded attribution. The one-third benefit allocation to the CGIAR research program in maize was assumed after discussing with breeders given data limitations, and similar benefit sharing assumptions were used in previous impact studies, for example, Evenson and Gollin (2003). In our case, while about 80% of the parentage share of CGIAR-related varieties consists of older CGIAR varieties, the complementary activities of NARS partners and private sector companies – identifying the varietal traits to target in the breeding, facilitating the partnership with CGIAR and other national partner institutions, developing congenial agronomic practices, popularizing varieties among maize farmers, etc. – are instrumental in generating the observed effect in national maize production. Having said that, the effect of these activities is not easy to quantify; data on investments made by the NARS and the private sector in maize breeding and agronomy are largely unavailable. While we followed an equal sharing of estimated benefits across R&D partners, a sensitivity analysis was additionally conducted by taking 25% and 75% benefit allocation for the CGIAR maize breeding program. The findings are provided in Appendix V.
