# Supplementary material for: Impact of CGIAR maize germplasm in Sub-Saharan Africa
Source: Field Crops Res. 2023 Jan 1;290:108756. doi: 10.1016/j.fcr.2022.108756 (PMC9760565; doi:10.1016/j.fcr.2022.108756)
Supplement: Supplementary file 1 — Supplementary material [file mmc1.docx]

**Supplementary Materials, S1**

**S1.1:** Data sources for varietal adoption estimates

**S1.2**: Adoption data sources of open-pollinated varieties (OPVs) and hybrids

|  | Number of adoption-datapoints obtained from different sources | | | | |
| --- | --- | --- | --- | --- | --- |
|  | Expert survey | Literature review | Seed sales registry | Unpublished reports / datasets | Others |
| OPV maize | 104  (11.8%) | 274  (31.1%) | 40  (4.5%) | 464  (52.6%) | 0  (0.0%) |
| Hybrid maize | 58  (8.7%**) | 140  (21.0%***) | 140  (21.0%***) | 318  (47.8%*) | 10  (1.5%***) |

Notes: Figures in parentheses show the % of row totals. *, **, ***: The difference in the percentage figures between OPVs and hybrids is statistically significant at 0.10, 0.05, and 0.01 levels respectively.

Of the total of 1,548 datapoints obtained at the variety-level (CGIAR and non-CGIAR varieties), 27% were from published literature, 50% from unpublished project documents, 12% from seed registries, and 10% from expert surveys conducted by authors. The adoption datapoints on OPVs were significantly higher in number (+32%) than hybrids (S1.2), corresponding to the relative adoption of hybrids (36% during 2010-2015 in the study countries) and OPVs (64%) in farmers’ field. The adoption figures on OPVs relied more on unpublished data and literature review, whereas those on hybrids relied on unpublished reports and seed sales registries. The published literature reviewed includes: de Groote et al. 2015; Kassie et al. 2012; Morris et al. 2003; Lopez-Pereira and Morris 1994; Abate et al. 2017; Bosco 2016; Erenstein et al. 2011; Etoundi and Dia 2008; Kassie et al. 2017; Holden and Mangisoni 2013; Kiakanua et al. 2014; Chikobvu et al. 2014; da Luz Quinhentos et al. 2014; Mafouasson et al. 2020; Ragasa et al. 2013; Mahoussi et al. 2017; Tandzi et al. 2015; Tripp and Ragasa 2015.

**List of published papers**

1. Abate, Tsedeke; Fisher, Monica; Abdoulaye, Tahirou; Kassie, Girma T.; Lunduka, Rodney; Marenya, Paswel; Asnake, Woinishet (2017): Characteristics of maize cultivars in Africa: How modern are they and how many do smallholder farmers grow? In *Agriculture & Food Security* 6, pp. 1–17. DOI: 10.1186/s40066-017-0108-6.
2. Bosco, N. J. (2016): Farmers' perceptions on maize varieties in Rwanda: A case study of smallholder farmers in Rwimbogo sector in Gatsibo District. In *International Academic Journal of Innovative Research* 3 (9), pp. 1–8.
3. Chikobvu, S.; Kassie, Girma T.; Lunduka, R. W. (2014): Country Report: DT Maize Adoption Monitoring Survey, Zimbabwe. Mexico, D.F.: CIMMYT.
4. da Luz Quinhentos, M.; Lunduka, Rodney W.; Kandiwa, Vongai (2014): Country Report: DT Maize Adoption Monitoring Survey, Mozambique. Mexico, D.F.: CIMMYT.
5. de Groote, Hugo; Gitonga, Z.; Mugo, S.; Walker, T. S. (2015): Assessing the effectiveness of maize and wheat improvement from the perspectives of varietal output and adoption in East and Southern Africa. In Thomas S. Walker, Jeffrey Alwang (Eds.): Crop improvement, adoption, and impact of improved varieties in food crops in sub-Saharan Africa. Oxon, UK, Cambridge, USA: CABI Publishing, pp. 206–227.
6. Erenstein, Olaf; Kassie, Girma T.; Langyintuo, Augustine S.; Mwangi, W. M. (2011): Characterization of Maize Producing Households in Drought Prone Regions of Eastern Africa. Mexico, D.F.: CIMMYT (CIMMYT Socio-Economics Working Paper, 1).
7. Etoundi, S.M.N.; Dia, B. K. (2008): Determinants of the adoption of improved varieties of Maize in Cameroon: Case of CMS 8704. Proceedings of the African Rconomic Conference, Tunis, Tunisia, 12-14 November 2008, pp. 397-413.
8. Holden, Stein T.; Mangisoni, Julius H. (2013): Input subsidies and improved maize varieties in Malawi: What can we learn from the impacts in a drought year? Center for Land Tenure Studies Working Paper 07/13. Ås, Norway: Norwegian University of Life Sciences (NMBU).
9. Kassie, Girma T.; Abdulai, Awudu; Greene, William H.; Shiferaw, Bekele; Abate, Gashaw Tadesse; Tarekegne, Amsal; Sutcliffe, Chloe (2017): Modeling preference and willingness to pay for drought tolerance (DT) in maize in Rural Zimbabwe. In *World Development* 94, pp. 465–477. DOI: 10.1016/j.worlddev.2017.02.008.
10. Kassie, Girma Tesfahun; Erenstein, O.; Mwangi, W.; La Rovere, Roberto; Setimela, Peter; Langyintuo, Augustine (2012): Characterization of Maize Production in Southern Africa: Synthesis of CIMMYT/DTMA Household Level Farming System Surveys in Angola, Malawi, Mozambique, Zambia and Zimbabwe. Mexico, D.F.: CIMMYT.
11. Kiakanua, M.; Dongala, K.; Pedro, I. C.; Morais, O.; Kassie, Girma T.; Nzambi, V. K. et al. (2014): Country Report: DT Maize Adoption Monitoring Survey, Angola. Mexico, D.F.: CIMMYT.
12. Lopez-Pereira, M. A.; Morris, M. L. (1994): Impacts of International Maize Breeding Research in the Developing World, 1966-1990. Mexico, D.F.: International Maize and Wheat Improvement Center (CIMMYT).
13. Mafouasson, Hortense Noëlle Apala; Kenga, Richard; Gracen, Vernon; Ntsomboh-Ntsefong, Godswill; Tandzi, Liliane Ngoune; Tata Ngome, Precillia Ijang (2020): Production constraints, farmers’ preferred characteristics of maize varieties in the bimodal humid forest zone of Cameroon and their implications for plant breeding. In *Agricultural Research* 9 (4), pp. 497–507. DOI: 10.1007/s40003-020-00463-6.
14. Mahoussi, F. E.; Adegbola, Patrice Ygue; Zannou, A.;. Hounnou, E. F.; Biaou, G. (2017): Adoption assessment of improved maize seed by farmers in Benin Republic. In *Journal of Agricultural and Crop Research* 5 (3), pp. 32–41.
15. Morris, M.; Mekuria, M.; Gerpacio, R. (2003): Impacts of CIMMYT maize breeding research. In R. E. Evenson, D. Gollin (Eds.): Crop Variety Improvement and its Effect on Productivity: The Impact of International Agricultural Research. Oxon, UK, Cambridge, USA: CABI Publishing, pp. 135–158.
16. Ragasa, C.; Dankyi, A.; Acheampong, P.; Wiredu, A. N.; Chapoto, A.; Asamoah, M.; Tripp, R. (2013): Patterns of adoption of improved maize technologies in Ghana. Ghana Strategy Support Program Working Paper 36. Accra, Ghana: International Food Policy Research Institute (IFPRI). Available online at http://www.ifpri.org/publication/patterns-adoption-improved-maize-technologies-ghana, checked on 7/7/2022.
17. Tandzi, Liliane Ngoune; Ngonkeu, Eddy M.; Nartey, E.; Yeboah, M.; Ngeve, Jacob; Mafouasson, Hortense Noëlle Apala et al. (2015): Farmers' adoption of improved maize varieties in the humid forest area of Cameroon. In *International Journal of Scientific Engineering and Applied Science* 1 (8), pp. 17–28.
18. Tripp, Robert; Ragasa, Catherine (2015): Hybrid Maize Seed Supply in Ghana. GSSP Working Paper 40. Accra, Ghana: International Food Policy Research Institute (IFPRI).
