# Supplementary material for: Impact of CGIAR maize germplasm in Sub-Saharan Africa
Source: Field Crops Res. 2023 Jan 1;290:108756. doi: 10.1016/j.fcr.2022.108756 (PMC9760565; doi:10.1016/j.fcr.2022.108756)
Supplement: Supplementary file 2 — Supplementary material [file mmc2.docx]

**Supplementary Materials, S2**

**Adoption of hybrid maize in the study countries**

|  | Weighted mean adoption rate of hybrid maize, which shows the share of national maize area | | | |
| --- | --- | --- | --- | --- |
|  | 1995-1999 | 2000-2004 | 2005-2009 | 2010-2015 |
| Angola | 0.00  (0.00) | 0.00  (0.00) | 0.00  (0.00) | 0.00  (0.00) |
| Benin | 0.00  (0.00) | 0.00  (0.00) | 0.00  (0.00) | 0.00  (0.00) |
| Cameroon | 0.01  (0.00) | 0.04  (0.01) | 0.09  (0.01) | 0.09  (0.00) |
| Ethiopia | 0.06  (0.01) | 0.17  (0.02) | 0.28  (0.01) | 0.61  (0.04) |
| Ghana | 0.00  (0.00) | 0.00  (0.00) | 0.00  (0.00) | 0.01  (0.00) |
| Guinea | 0.13  (0.02) | 0.26  (0.02) | 0.39  (0.02) | 0.34  (0.02) |
| Kenya | 0.18  (0.02) | 0.39  (0.04) | 0.72  (0.03) | 0.82  (0.01) |
| Madagascar | 0.00  (0.00) | 0.00  (0.00) | 0.00  (0.00) | 0.00  (0.00) |
| Malawi | 0.06  (0.01) | 0.22  (0.04) | 0.53  (0.06) | 0.77  (0.02) |
| Mali | 0.00  (0.00) | 0.00  (0.00) | 0.00  (0.00) | 0.00  (0.00) |
| Mozambique | 0.01  (0.00) | 0.04  (0.01) | 0.21  (0.02) | 0.30  (0.02) |
| Nigeria | 0.06  (0.01) | 0.11  (0.01) | 0.15  (0.01) | 0.11  (0.03) |
| Rwanda | 0.00  (0.00) | 0.00  (0.00) | 0.00  (0.00) | 0.20  (0.07) |
| Senegal | 0.00  (0.00) | 0.00  (0.00) | 0.00  (0.00) | 0.00  (0.00) |
| Tanzania | 0.01  (0.00) | 0.04  (0.01) | 0.15  (0.02) | 0.39  (0.03) |
| Uganda | 0.01  (0.00) | 0.03  (0.01) | 0.15  (0.02) | 0.39  (0.03) |
| Zambia | 0.05  (0.01) | 0.23  (0.05) | 0.61  (0.05) | 0.76  (0.01) |
| Zimbabwe | 0.04  (0.01) | 0.29  (0.05) | 0.66  (0.05) | 0.90  (0.02) |
| *Overall* | ***0.05***  ***(0.01)*** | ***0.13***  ***(0.01)*** | ***0.27***  ***(0.03)*** | ***0.36***  ***(0.03)*** |

Note: Figures in parentheses show the standard error of the mean values across years. The annual maize area was used as the analytical weight for aggregation.
