# Supplementary material for: Impact of CGIAR maize germplasm in Sub-Saharan Africa
Source: Field Crops Res. 2023 Jan 1;290:108756. doi: 10.1016/j.fcr.2022.108756 (PMC9760565; doi:10.1016/j.fcr.2022.108756)
Supplement: Supplementary file 3 — Supplementary material [file mmc3.docx]

**Supplementary Materials 3**. Effect of estimated farmer adoption of CGIAR germplasm on maize yield at the national level in the study countries in SSA – alternative model specifications.

|  | Whole sample | | |  | Reduced sample (excluding years when varieties with unknown parentage are >10%) | | |
| --- | --- | --- | --- | --- | --- | --- | --- |
|  | OLS | Fixed Effects | DPD |  | OLS | Fixed Effects | DPD |
| *Effect during the entire study period (1995-2015)* |  |  |  |  |  |  |  |
| Estimated adoption of new CGIAR varieties, released in 1995 or later (share of national maize area, 0-1) | 0.994^***^  (0.312) | 0.922^***^  (0.281) | 0.779^***^ (0.190) |  | 1.150^***^  (0.245) | 1.046^***^  (0.309) | 0.810^***^ (0.209) |
| *Square of* estimated adoption of new CGIAR varieties | -0.693^***^  (0.379) | -0.639^**^ (0.311) | -0.718^***^  (0.215) |  | -0.878^***^  (0.345) | -0.756^**^  (0.332) | -0.762^***^  (0.227) |
| Estimated adoption of old CGIAR varieties, released before 1995 (share of national maize area, 0-1) | 0.410^***^  (0.119) | -0.176  (0.175) | 0.011  (0.109) |  | 0.414^***^  (0.115) | -0.756  (0.332) | 0.080  (0.119) |
| Other controls used | Yes | Yes | Yes |  | Yes | Yes | Yes |
| Number of observations | 378 | 378 | 342 |  | 357 | 357 | 321 |
| F statistics / Wald Chi^2^ | 26.65^***^ | 17.83^***^ | 505.50^***,a^ |  | 34.10^***^ | 17.02^***^ | 529.20^***,a^ |
| *Effect during 2006-2015* |  |  |  |  |  |  |  |
| Estimated adoption of new CGIAR varieties, released in 1995 or later (share of national maize area, 0-1) | 1.617^***^  (0.440) | 0.943^**^  (0.451) | 0.689^**^  (0.308) |  | 1.887^***^  (0.449) | 1.665^***^  (0.594) | 1.135^***^  (0.380) |
| *Square of* estimated adoption of new CGIAR varieties | -1.482^***^  (0.519) | -0.582  (0.451) | -0.609^**^  (0.321) |  | -1.768^***^  (0.529) | -1.219^**^  (0.583) | -1.029^***^  (0.378) |
| Estimated adoption of old CGIAR varieties, released before 1995 (share of national maize area, 0-1) | 0.296^***^  (0.151) | -0.038  (0.295) | 0.234  (0.153) |  | 0.197  (0.158) | 0.056  (0.333) | 0.264  (0.170) |
| Other controls used | Yes | Yes | Yes |  | Yes | Yes | Yes |
| Number of observations | 12.49^***^ | 4.84^***^ | 204.04^***,a^ |  | 14.53^***^ | 4.63^***^ | 218.02^***,a^ |
| F statistics / Wald Chi^2^ | 180 | 180 | 180 |  | 159 | 159 | 159 |

*Notes*: The dependent variable is natural logarithm of the annual national maize yield (kg ha^-1^). ^***^: p≤0.01, ^**^ : p≤0.05. ^a^ Wald Chi2 statistic. Model with the shaded area is used to generate Figure 3. OLS stands for Ordinary Least Squares and DPD for Dynamic Panel Data model.
