# Supplementary material for: Impact of CGIAR maize germplasm in Sub-Saharan Africa
Source: Field Crops Res. 2023 Jan 1;290:108756. doi: 10.1016/j.fcr.2022.108756 (PMC9760565; doi:10.1016/j.fcr.2022.108756)
Supplement: Supplementary file 4 — Supplementary material [file mmc4.docx]

**Supplementary Materials, S4**

**Post-2015 developments in CGIAR-related maize breeding**

Accelerated replacement of old climate-vulnerable varieties with new resilient ones by farming communities is critical to climate adaptation efforts. In this context, SSA has seen substantive development and wider diffusion of stress-tolerant maize varieties after 2015 (Prasanna et al., 2021). Besides the shift of CGIAR breeding focus from yield enhancement to resilience building and yield maintenance, the centers’ initiatives to partner with SMEs for seed multiplication and dissemination helped realize a transformation over the past several years. CIMMYT and IITA have supported seed company partners in SSA by supplying breeder and the pre-basic seed of CGIAR-related maize varieties and technical backstopping on seed production since the early 2000s. The institutional support for the SME seed business has been augmented since 2007, and more recently, efforts on Quality Assurance/Quality Control, market segmentation, and territory planning, especially for SME seed companies, were introduced (Prasanna et al., 2021).

Based on a survey of maize cultivars from 13 countries of SSA, Abate et al. (2017) estimated the area-weighted average age (AWAA) of maize varieties in eastern, southern, and western Africa as 13.8, 15.4, and 16.4 years, respectively. Recently, Chivasa et al. (2022) estimated the AWAA of CIMMYT-related improved maize varieties in eastern and southern Africa to be 10.32 and 9.78 years, respectively. This significant decrease in the overall AWAA in eastern and southern Africa from 14 years in 2014 to 10 years in 2021 was attributed to the strengthening of seed systems, including intensive deployment of varieties with better genetics through effective and inclusive public-private partnerships (Prasanna et al. 2021; Chivasa et al. 2022).

Maize yields in the tropical rainfed environments are increasingly vulnerable to various climate-induced stresses, especially drought, heat, waterlogging, cold, diseases, parasitic weeds, and insect pests, which often come in combinations to severely impact maize crops. CGIAR, in partnership with several public and private sector institutions, has increased the intensity of engagement in breeding elite tropical maize germplasm with tolerance to key abiotic and biotic stresses, using an extensive managed stress screening network and on-farm testing system (Edmeades et al. 2017; Yacoubou et al. 2021; Prasanna et al. 2021). There is strong evidence in the literature that the stress-tolerant maize varieties released in collaboration between CGIAR, national breeding programs, and the private seed sector stabilize production, reduce downside risk, and improve livelihoods in tropical and subtropical production environments of SSA (Wossen et al. 2017; Amondo et al. 2019; Lunduka et al. 2019; Martey et al. 2020; Simtowe and Groote 2020; Katengeza and Holden 2021). The climate resilience of CGIAR-related maize varieties was evident during the 2015/2016 El Niño-affected season, when CIMMYT-derived drought-tolerant varieties yielded twice as much as major commercial ones when 40 maize hybrids were evaluated in 30 on-farm trials across Zambia, Zimbabwe, Malawi, and Mozambique (Setimela et al. 2018).

The CGIAR maize breeding programs also develop biofortified varieties, especially ProVA and kernel zinc enrichment (Prasanna et al., 2020). Several proVA-enriched maize varieties have been increasingly adopted in SSA during the last decade. In 2019, the certified seed production of IITA-derived proVA-enriched maize synthetics and hybrids was estimated to be 3,300 tons. They were marketed in Ghana, Mali, and Nigeria to cover about 148,000 ha. In 2019-20, over 1,270 tons of certified seed of 14 proVA-enriched maize hybrids were produced and commercialized in Zambia, Zimbabwe, Malawi, and Tanzania by 11 seed companies, covering an estimated 57,736 ha. Recent empirical studies have demonstrated that biofortified maize consumption could significantly benefit human health (Sheftel et al., 2017; Palmer et al., 2018). However, the research investment in QPM and other biofortified maize varieties has been declining over time, despite the increasing importance of alleviating malnutrition in continents like Africa. Possibly because of this reason, the adoption of QPM varieties has been only marginal in study countries during the 1995-2015 period (with the exception of Ghana, where an old QPM variety called *Obatampa* is still popular).

Despite the pronounced benefit-cost ratio of CGIAR R&D programs, more effort is required in order to reach smallholder farmers of Africa with climate-resilient and nutritionally enriched maize varieties. Increasing the genetic gains and delivering high-quality, climate-resilient, disease and insect-pest-resistant, and nutritionally enriched maize seed is more critical than ever to ensure food and nutritional security. This goal can be achieved in the coming years by (1) co-designing and implementing impactful breeding programs with NARS and SME partners in the seed sector to achieve synergies and enhance the sustainability of the genetic gains, (2) increased focus on nutritional quality and end-user-preferred traits that add value to the product profiles, (3) implementing continuous improvement plans to enhance breeding efficiency and to increase genetic gains in breeding pipelines in the target market segments, (4) further strengthening the maize seed systems, including accelerated varietal turnover and translation of increased genetic gains from on-station to on-farm, through unique public-private sector partnerships, and (5) constant monitoring of the varietal development and seed dissemination programs to identify and rectify the institutional bottlenecks preventing smallholders adopting improved germplasm.

**References**

Abate, Tsedeke; Fisher, Monica; Abdoulaye, Tahirou; Kassie, Girma T.; Lunduka, Rodney; Marenya, Paswel; Asnake, Woinishet (2017): Characteristics of maize cultivars in Africa: How modern are they and how many do smallholder farmers grow? In *Agriculture & Food Security* 6, Article 30, pp. 1–17. DOI: 10.1186/s40066-017-0108-6.

Amondo, Emily; Simtowe, Franklin; Rahut, Dil Bahadur; Erenstein, Olaf (2019): Productivity and production risk effects of adopting drought-tolerant maize varieties in Zambia. In *International Journal of Climate Change Strategies and Management* 11 (4), pp. 570–591. DOI: 10.1108/IJCCSM-03-2018-0024.

Chivasa, Walter; Worku, Mosisa; Teklewold, Adefris; Setimela, Peter; Gethi, James; Magorokosho, Cosmos et al. (2022): Maize varietal replacement in Eastern and Southern Africa: Bottlenecks, drivers and strategies for improvement. In *Global Food Security* 32, p. 100589. DOI: 10.1016/j.gfs.2021.100589.

Edmeades, G. O.; Trevisan, Walter; Prasanna, Boddupalli M.; Campos, Hugo (2017): Tropical Maize (Zea mays L.). Chapter 3. In Hugo Campos, Peter D.S. Caligari (Eds.): Genetic Improvement of Tropical Crops. Cham: Springer International Publishing, pp. 57–109.

Katengeza, Samson P.; Holden, Stein T. (2021): Productivity impact of drought tolerant maize varieties under rainfall stress in Malawi: A continuous treatment approach. In *Agricultural Economics* 52 (1), pp. 157–171. DOI: 10.1111/agec.12612.

Lunduka, Rodney Witman; Mateva, Kumbirai Ivyne; Magorokosho, Cosmos; Manjeru, Pepukai (2019): Impact of adoption of drought-tolerant maize varieties on total maize production in southeastern Zimbabwe. In *Climate and Development* 11 (1), pp. 35–46. DOI: 10.1080/17565529.2017.1372269.

Martey, Edward; Etwire, Prince M.; Kuwornu, John K.M. (2020): Economic impacts of smallholder farmers’ adoption of drought-tolerant maize varieties. In *Land Use Policy* 94, p. 104524. DOI: 10.1016/j.landusepol.2020.104524.

Palmer, Amanda C.; Craft, Neal E.; Schulze, Kerry J.; Barffour, Maxwell; Chileshe, Justin; Siamusantu, Ward; West, Keith P. (2018): Impact of biofortified maize consumption on serum carotenoid concentrations in Zambian children. In *European Journal of Clinical Nutrition* 72 (2), pp. 301–303. DOI: 10.1038/s41430-017-0054-1.

Prasanna, Boddupalli M.; Cairns, Jill E.; Zaidi, P. H.; Beyene, Yoseph; Makumbi, Dan; Gowda, Manje et al. (2021): Beat the stress: Breeding for climate resilience in maize for the tropical rainfed environments. In *Theoretical and Applied Genetics* 134 (6), pp. 1729–1752. DOI: 10.1007/s00122-021-03773-7.

Prasanna, Boddupalli M.; Palacios-Rojas, Natalia; Hossain, Firoz; Muthusamy, Vignesh; Menkir, Abebe; Dhliwayo, Thanda et al. (2020): Molecular breeding for nutritionally enriched maize: Status and prospects. In *Frontiers in Genetics* 10, p. 1392. DOI: 10.3389/fgene.2019.01392.

Setimela, Peter; Gasura, Edmore; Thierfelder, Christian; Zaman-Allah, Mainassara; Cairns, Jill E.; Boddupalli, Prasanna M. (2018): When the going gets tough: Performance of stress tolerant maize during the 2015/16 (El Niño) and 2016/17 (La Niña) season in southern Africa. In *Agriculture, Ecosystems & Environment* 268, pp. 79–89. DOI: 10.1016/j.agee.2018.09.006.

Sheftel, Jesse; Gannon, Bryan M.; Davis, Christopher R.; Tanumihardjo, Sherry A. (2017): Provitamin A-biofortified maize consumption increases serum xanthophylls and 13C-natural abundance of retinol in Zambian children. In *Experimental Biology and Medicine* 242 (15), pp. 1508–1514. DOI: 10.1177/1535370217728500.

Simtowe, Franklin; Groote, Hugo de (2020): Seasonal participation in maize markets in Zambia: Do agricultural input subsidies and gender matter? In *Food Security*. DOI: 10.1007/s12571-020-01106-y.

Wossen, Tesfamicheal; Abdoulaye, Tahirou; Alene, Arega; Feleke, Shiferaw; Menkir, Abebe; Manyong, Victor (2017): Measuring the impacts of adaptation strategies to drought stress: The case of drought tolerant maize varieties. In *Journal of Environmental Management* 203 (Pt 1), pp. 106–113. DOI: 10.1016/j.jenvman.2017.06.058.

Yacoubou, Abdoul-Madjidou; Zoumarou Wallis, Nouhoun; Menkir, Abebe; Zinsou, Valerien A.; Onzo, Alexis; Garcia-Oliveira, Ana Luísa et al. (2021): Breeding maize (Zea mays) for Striga resistance: Past, current and prospects in sub-saharan africa. In *Plant Breeding* 140 (2), pp. 195–210. DOI: 10.1111/pbr.12896.
